# Near work, outdoor activity, and myopia in children in rural China: the Handan offspring myopia study

**DOI:** 10.1186/s12886-017-0598-9

**Published:** 2017-11-17

**Authors:** Zhong Lin, Tie Ying Gao, Balamurali Vasudevan, Kenneth J. Ciuffreda, Yuan Bo Liang, Vishal Jhanji, Su Jie Fan, Wei Han, Ning Li Wang

**Affiliations:** 1 The Eye Hospital, School of Ophthalmology and Optometry, Wenzhou Medical University, No. 270 West College Road, Wenzhou, Zhejiang, 325027 China; 2 Handan Eye Hospital, Handan, Hebei China; 3 College of Optometry, Mid Western University, Glendale, AZ USA; 4 Department of Biological and Vision Sciences, SUNY College of Optometry, New York, NY USA; 5 Department of Ophthalmology, University of Pittsburgh School of Medicine, Pittsburgh, PA USA; 6 Department of Ophthalmology and Visual Sciences, The Chinese University of Hong Kong, Hong Kong, China; 7 Beijing Tongren Eye Center, Beijing Tongren Hospital, Capital Medical University, Beijing, China; 8 Centre for Public Health, School of Medicine, Dentistry and Biomedical Sciences, Queen’s University, Belfast, UK

**Keywords:** near work, outdoor activity, myopia, rural

## Abstract

**Background:**

The near work and outdoor activity are the most important environmental risk factors for myopia. However, data from Chinese rural children are relatively rare and remain controversial. Therefore, the purpose of this study was to evaluate the relationship of both near work and outdoor activities with refractive error in rural children in China.

**Methods:**

In this cross-sectional study, 572 (65.1%) of 878 children (6–18 years of age) were included from the Handan Offspring Myopia Study (HOMS). Information from the parents on these children, as well as the parent’s non-cycloplegic refraction, were obtained from the database of the Handan Eye Study conducted in the years 2006–2007. A comprehensive vision examination, including cycloplegic refraction, and a related questionnaire, were assessed on all children.

**Results:**

The overall time spent on near work and outdoor activity in the children was 4.8 ± 1.6 and 2.9 ± 1.4 h per day, respectively. Myopic children spent more time on near work (5.0 ± 1.7 h vs.4.7 ± 1.6 h, *p* = 0.049), while no significant difference was found in outdoor activity hours (2.8 ± 1.3 h vs. 3.0 ± 1.4 h, *p* = 0.38), as compared to non-myopic children. In the multiple logistic analysis, in general, no association between near work and myopia was found after adjusting for the children’s age, gender, parental refractive error, parental educational level, and daily outdoor activity hours [odds ratio (OR), 95% confidence interval (CI): 1.10, 0.94–1.27]. However, a weak protective effect of the outdoor activity on myopia was found (OR, 95% CI: 0.82, 0.70–0.96), after adjusting for similar confounders.

**Conclusions:**

In general, no association between near work and myopia was found, except for the high near work subgroup with moderate outdoor activity levels. A weak protective effect of outdoor activity on myopia in Chinese rural children was observed.

**Electronic supplementary material:**

The online version of this article (10.1186/s12886-017-0598-9) contains supplementary material, which is available to authorized users.

## Background

Myopia is a common vision disorder. The prevalence of myopia in Chinese children living in China [1] was reported to be higher compared to children from Nepal, [2, 3] India, [4, 5] Singapore, [6, 7] Africa, [8] Chile, [9] Australia, [10, 11] the United States, [12] and England [13, 14].

Cross-sectional and longitudinal studies on Chinese urban children have found an association between near work/outdoor activity and myopia/myopic progression. [15–24] Regarding Chinese rural children, Lu et al. [25] reported no association between either outdoor activity or near work with myopia in the Xichang Pediatric Refractive Error Study (X-PRES) in southern rural China. In contrast, Wu et al. reported that more frequent outdoor activity was associated with a lower prevalence of myopia in Taiwanese rural Chinese primary school children [26]. Interestingly, data from Chinese rural children are relatively rare and remain controversial.

Therefore, we conducted the Handan Offspring Myopia Study (HOMS), which aimed to assess the relationship between near work/outdoor activity and myopia in a rural population in northern China.

## Methods

The HOMS, an offspring study of the Handan Eye Study (HES), primarily aimed to investigate the familial associations for myopia among parents and their offspring aged 6 to 18 years in rural northern China, as well as to assess the myopic shift between the two generations and its putative risk factors [27]. Subjects were drawn from the HOMS, which is a part of the offspring of HES [28]. The study design, procedures, and characteristics of HOMS are reported elsewhere [27]. In brief, the study was undertaken in a rural population in Yongnian County, Handan, which is located in southern Hebei province (about 500 km south of Beijing). This geographic area has demographic characteristics similar to other rural regions of China according to the 2000 National Census [28]. Thirteen villages were randomly selected for the HES. Among them, 6 villages with age of parents more than 30 years were selected for the HOMS. From March to June in 2010, 878 of 1238 children eligible for the HOMS (70.9% response rate), aged 6 to 18 years, were examined in the HOMS. There were 462 boys (52.6%) and 416 girls (47.4%), aged 10.4 ± 2.4 and 10.8 ± 2.6 years, respectively. All participants were self-identified Han people. In contrast to the non-participants, children who participated were more likely to be boys, younger, and studying or working near the villages. Information relating to the parents was obtained from the HES.

This study adhered to the Declaration of Helsinki. Written, informed consent was obtained from at least one parent/guardian. Ethics Committee approval was obtained from the Handan Eye Hospital.

### Questionnaire

Each participant completed a standard, myopia-based questionnaire in a face-to-face interview by a trained staff member conducted in the local dialect of the study site. This questionnaire was used in the Sydney Myopia Study, and it was slightly modified and translated into Chinese [18, 20, 29]. The interview covered a broad range of questions regarding various daily activities. These activities were classified into near work, midworking distance, and outdoor activities. Near work activities were defined as those having less than a 50 cm working distance, including drawing pictures, doing homework, reading books, attending additional classes, and using handheld computers. Activities at the midworking distance included watching television, playing video games and using computers. Outdoor activities included leisure time spent outside (e.g., staying in the backyard, walking, riding a bike/scooter, going shopping) and outdoor sports (e.g., running, playing ball, skipping rope). Activity levels were graded as low, moderate, and high using population tertiles of the average daily hours spent on these different activities.

### Cycloplegic refraction

Refraction was performed using an autorefractor (model KR8800, Topcon, Tokyo, Japan) before and after cycloplegia in the children. Cycloplegic autorefraction was performed 20 min after instilling 3 drops of cyclopentolate 1% (Cyclogyl, Alcon).Three readings were obtained in each eye, and the average was recorded. A fully dilated pupil was defined as one with a diameter of ≥6 mm and having absence of any pupillary light reflex.

Information relating to the parents was obtained from the HES database including non-cycloplegic autorefracion. All examinations were performed using the same protocols and equipment as the HES [30].

### Data analysis and definitions

No imputations were done for the missing data. Only data from the right eye were used, since there was a high correlation in spherical equivalent (SE) between the right and left eye (*r*_pearson_ = 0.94, *p* < 0.001). Data were analyzed using commercial software (SAS ver. 9.1.3; SAS Institute, Cary, NC) with the significance level set at less than 0.05.

The SE was calculated as the sphere +1/2 cylinder. Myopia, emmetropia, and hyperopia were defined as SE < −0.5 diopters (D), −0.5D ≤ SE ≤0.5D, and SE > 0.5D, respectively [27, 30]. Average parental refractive error was defined as the combined average of the non-cycloplegic SE of the father and mother. Average daily hours of activities were presented as the mean ± standard deviation. The daily hours of activities were calculated using the formula: [(average hours spend on weekday) × 5 + (average hours spend on weekends) × 2)]/7. Diopter-hours were calculated using a cumulative near work exposure variable at the near and midworking distances using the formula: 3 × (reading for pleasure hours +study hours) + 2 × (computer hours + video games hours) + watching television hours [18, 20]. Activity level was first analyzed continuously as the average daily hours, and then by tertile activity groups. Generalized estimating equations (GEEs) were used to assess the association between the SE and daily activity/confounders (fixed effects), as well as considering the children from the same family (family effect) as a random effect. Parental educational level was categorized as the following: illiterate, primary school, junior high school, and senior high school and above. The joint effect of near work and outdoor activities, as well as family effect, using stepwise logistic regression models was performed after adjusting for the different risk factors using GEEs. Odds ratios (OR) and 95% confidence intervals (CIs) are presented.

## Results

**Fig. 1:**
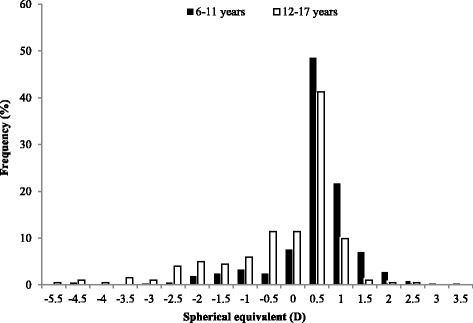
Distribution of refractive error in children 6–11 years old and 12–17 years old

A total of 878 (70.9%) of 1238 children aged 6 to 18 years participated in this study. Of these, 598 with completed cycloplegic autorefraction, myopia questionnaire, and parental refractive information were included. Overall, 13 children with either amblyopia or strabismus, 1 child with previous corneal surgery, and 12 children’s parents with either amblyopia or strabismus were excluded. Hence, 572 (65.1%) of 878 children were included in the final analysis. There were 170, 132, 42, and 3 families with one, two, three, and four child(ren). No significant difference was found for the children’s age, prevalence of myopia, and SE between the included and excluded children (*p* = 0.44, *p* = 0.65, and *p* = 0.63, respectively). However, there were more boys in the included children as compared to the excluded children (*p* = 0.03) (Table 1). Fig. 1 presents the distribution of refractive error in children 6–11 years old and 12–17 years old.

**Table 1:** Characteristics of the included and excluded children

	Included (*N* = 572)	Excluded (*N* = 306)	*P*
Age (years)	10.6 ± 2.5	10.5 ± 2.5	0.44
Gender (boys:girls)	316:256	146:160	0.03
Prevalence of myopia (%)^a^			
Boys	17.1	17.2	0.86
Girls	32.8	27.3	0.12
Total	24.1	22.6	0.65
Spherical equivalent (D)^a^			
Boys	0.15 ± 1.18	0.19 ± 1.44	0.81
Girls	−0.17 ± 1.27	−0.24 ± 1.75	0.65
Total	0.01 ± 1.23	−0.05 ± 1.63	0.63

^a^Refractive error data were missing in 24 boys and 17 girls in the excluded group

Children spent 4.8 ± 1.6 and 2.9 ± 1.4 h per day on near work and outdoor activity, respectively. Regarding the daily near work time in hours, boys and myopic children spent significantly more time on near work than either girls (4.9 ± 1.7 vs. 4.6 ± 1.5, *p* = 0.042) or non-myopic children (5.0 ± 1.7 vs. 4.7 ± 1.6, *p* = 0.049), respectively. Although there were significant differences among the paternal educational levels for near work time (*p* = 0.018), no significant differences were found in the subsequent pair-wise comparisons. No significant differences were found among the other groups, e.g., number of myopic parents, maternal educational levels (Table 2). Regarding the daily outdoor activity time, only a borderline difference was found among the maternal education levels (*p* = 0.054). No significant differences were found among the other groups for outdoor activity. The daily hours spent on mid-work distance activities were also not associated with the mean SE (*r*_spearman_ = −0.003, *p* = 0.94).

**Table 2:** Near work and outdoor activity time (hours per day) in Handan Offspring Myopia Study children

	N	Near work		Outdoor	
		Mean ± SD	*p* value	Mean ± SD	*p* value
Gender					
Boys	316	4.9 ± 1.7	0.042^a^	3.0 ± 1.4	0.12^a^
Girls	256	4.6 ± 1.5		2.8 ± 1.4	
Refractive Status					
Myopia	138	5.0 ± 1.7	0.049^a^	2.9 ± 1.3	0.38^a^
No myopia	434	4.7 ± 1.6		3.0 ± 1.4	
Number of myopic parents					
None	197	4.6 ± 1.7	0.32^b^	2.9 ± 1.3	0.74^b^
Either	272	4.9 ± 1.7		2.9 ± 1.4	
Both	103	4.7 ± 1.4		3.0 ± 1.5	
Maternal education levels					
Illiteracy	87	4.7 ± 1.6	0.17^b^	2.9 ± 1.2	0.054^b^
Primary school	279	4.6 ± 1.7		2.8 ± 1.3	
Junior high school	189	5.0 ± 1.5		3.2 ± 1.6	
Senior high school and above	17	4.8 ± 1.6		3.1 ± 1.4	
Paternal education levels					
Illiteracy	21	5.4 ± 2.5	0.018^b^	3.0 ± 1.1	0.38^b^
Primary school	166	4.6 ± 1.6		2.8 ± 1.4	
Junior high school	343	4.7 ± 1.5		3.0 ± 1.5	
Senior high school and above	42	5.3 ± 2.1		2.8 ± 1.3	
Total	572	4.8 ± 1.6		2.9 ± 1.4	

^a^t-test

^b^generalized linear models

**Fig. 2:**
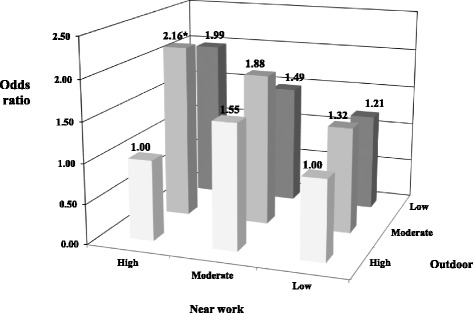
Multivariable-adjusted odds ratios (adjusted for children’s age, gender, average parental refractive error, maternal and paternal education level) for myopia by reported average daily hours spent on near work versus outdoor activities. Activities were divided into tertiles of high, moderate, and low levels of activity. The group with high levels of outdoor activity and low levels of near work is the reference group. The subgroup with high near work levels and moderate outdoor levels was significantly at risk for myopes (asterisk)

Table 3 and Table 4 present the mean SE of the children and their associations with daily activity hours, after being divided into tertile groups among the different subgroups. A high myopic refractive error was found in the fathers with the primary school education level subgroup if their children spent more time on near work (*p* = 0.01). The combined effects of outdoor and near work activities on the odds for myopia are presented in Fig. 2. Children with a high level of outdoor activity and low level of near work were used as the reference group (OR 1.0). Children with moderate outdoor activity and high near work had higher odds for myopia than the reference group (OR, 2.16; 95% CI, 0.69–6.77). However, none of the other subgroups had significant odds for myopia compared to the reference group (Fig. 2).

**Table 3:** Mean spherical equivalent (diopter)^a^ as a function of near work activity (tertiles of hours per day)

Near work activity (hours per day)^b^	Low (0~4.0) *N* = 185	Moderate (4.0~5.1) *N* = 196	High (>5.1) *N* = 191	*P* value
Gender				
Boys	0.33	0.08	0.06	0.17
Girls	−0.15	−0.19	−0.20	0.96
Refractive Status				
Myopia	−1.73	−1.74	−1.80	0.95
No myopia	0.63	0.52	0.53	0.25
Number of myopic parents				
None	0.09	−0.04	0.04	0.80
Either	0.30	0.04	0.01	0.17
Both	−0.29	−0.32	−0.46	0.89
Maternal education level				
Illiteracy	0.15	0.19	0.12	0.97
Primary school	−0.01	−0.01	0.00	0.99
Junior high school	0.39^c^	−0.13	−0.04	0.06
Senior high school and above	−1.30	−0.68	−0.85	0.88
Paternal education level				
Illiteracy	0.08	0.27	0.53	0.50
Primary school	0.38^c^	−0.11	−0.15	0.01
Junior high school	−0.06	−0.05	0.04	0.78
Senior high school and above	0.11	−0.01	−0.61	0.50
Total	0.10	−0.04	−0.06	0.35

^a^Adjusted for children’s age, gender, average parental refractive error, maternal and paternal education level, and outdoor activity time as fixed effects, and family effect as a random effect

^b^Includes drawing, homework, reading, and handheld computer use. Cut-off points were based on population tertiles for average daily hours spent at near

^c^Significant (Bonferroni test) compared with the highest tertile of activity as the reference group

**Table 4:** Mean spherical equivalent (diopter)^a^ as a function of outdoor activity (tertiles of hours per day)

Outdoor activity (hours per day)^b^	Low (0~2.3) *N* = 191	Moderate (2.3~3.2) *N* = 187	High (>3.2) *N* = 194	*P* value
Gender				
Boys	0.05	0.10	0.27	0.32
Girls	−0.13	−0.30	−0.10	0.49
Refractive Status				
Myopia	−1.69	−1.76	−1.84	0.82
No myopia	0.54	0.53	0.61	0.52
Number of myopic parents				
None	0.03	−0.19	0.25	0.10
Either	0.08	−0.01	0.26	0.24
Both	−0.53	−0.09	−0.46	0.32
Maternal education level				
Illiteracy	0.16	−0.10	0.40	0.09
Primary school	−0.09	−0.05	0.14	0.37
Junior high school	0.01	−0.07	0.11	0.68
Senior high school and above	0.11	−0.94	−1.68	0.52
Paternal education level				
Illiteracy	−0.01	0.22	0.54	0.62
Primary school	0.07	−0.11	0.22	0.30
Junior high school	−0.06	−0.11	0.10	0.31
Senior high school and above	−0.27	−0.12	−0.25	0.97
Total	−0.04	−0.09	0.12	0.20

^a^Adjusted for children’s age, gender, average parental refractive error, maternal and paternal education level, and near work time as fixed effects, and family effect as a random effect

^b^Includes outdoor sports, playing out of doors, and other outdoor leisure activities. Cut-off points were based on population tertiles for average daily hours spent outside

Stepwise multiple logistic models were used to analyze the correlation between daily near work/ outdoor activity hours and myopia (Table 5). In a univariate model, children who spent more time on near work were 1.12 (95% CI 1.01–1.25) times more likely to be myopic. However, no significant association between daily near work hours and myopia was found after adjusting for confounders, such as the children’s age, gender, average parental refractive error, parental education level, and daily outdoor activity hours. Outdoor activity had no protective effect for myopia (OR, 0.93; 95% CI, 0.82–1.07) in the univariate model. However, after adjusting for children’s age, gender, average parental refractive error, parental education level, and daily near work hours, outdoor activity showed a weak protective effect for myopia (OR, 0.82; 95% CI, 0.70–0.96).

**Table 5:** Stepwise logistic analysis of daily near work and outdoor activity hours for myopia

	Near work		Outdoor	
	OR	95% CI	OR	95% CI
Model 1	1.12	1.01–1.25	0.93	0.82–1.07
Model 2	1.07	0.94–1.21	0.86	0.74–1.00
Model 3	1.05	0.92–1.21	0.86	0.75–1.00
Model 4	1.10	0.94–1.27	0.82	0.70–0.96

Model 1 for near work: adjusted for near work time as a fixed effects, and family effect as a random effect; Model 1 for outdoor: adjusted for outdoor activity time as fixed effect, and family effect as a random effect

Model 2: model 1 + children’s age and gender

Model 3: model 2 + average parental refractive error

Model 4 for near work: model 3 + maternal and paternal education level, and outdoor activity time; Model 4 for outdoor: model 3 + maternal and paternal education level, and near work time

## Discussion

Although cross-sectional and longitudinal studies on Chinese urban children have found an association between near work/outdoor activity and myopia/myopic progression, [15–20] studies related to the association of near work and outdoor activity with myopia are rare and equivocal among Chinese rural children [24]. In Taiwan, Wu et al. reported that more frequent outdoor activity was associated with a lower prevalence of myopia in rural Chinese primary school children [16, 26]. In contrast, in the Strabismus, Amblyopia and Refractive Error Study (STARS) in Singaporean preschool Chinese children, and in the X-PRES in Chinese rural teenagers, negative results were reported related to both near work and outdoor activity [25, 31]. A meta-analysis that included 7 cross-sectional studies (including STARS and X-PRES) have reported that one additional hour per week spent outdoors would reduce the odds by 2% (odds ratio, 95% confidence interval: 0.98, 0.97–0.99) of having myopia in children and adolescents.

The current study performed in Handan reports the effect and possible interaction of near work and outdoor activity on myopia in a wide age range among the Chinese rural population. In the present study, the parental refractive error, one of the important confounding factors for children’s myopia, [31, 32] was obtained directly. We found that myopic children spent more time on near work compared to non-myopic children. However, in general, the daily near work was not associated with the children’s myopia as per the multiple logistic analysis, after adjusting for the children’s age, gender, average parental refractive error, parental education level, and outdoor activity time. This was consistent with previous studies conducted in Caucasians, [23, 33, 34] and East Asians, living in Sydney [23]. This was also consistent with findings in Singaporean preschool Chinese children in STARS, and in X-PRES in Chinese rural teenagers [25, 31]. However, our results were different from those reported in Beijing urban students [16, 17, 20].

A weak protective effect of outdoor activity for myopia was found in the present study. The association between more time outdoors and either a lower prevalence of myopia, or more hyperopic refractive error, was reported in Caucasians [23, 34, 35] as well as Singaporean teenagers [24]. Furthermore, this association was also found in the Chinese [16, 17, 26]. In Taiwan, Wu et al. reported that more frequent outdoor activity was associated with a lower prevalence of myopia (OR, 0.3; 95% CI, 0.1–0.9) in rural Chinese school children aged 7–12 years [26]. Guo et al. also reported that less outdoor activity and more time spent indoors studying was associated with myopia progression and elongation of axial length in primary students in grades 1 and grade 4 in Beijing (age range: 5–13 years) [17]. However, the association was not found either in X-PRES in Chinese rural teenagers (mean age: 14.6 years) [25] or in another study involving Beijing urban school children (aged 6–17 years) [20]. It is noteworthy that the protective effect of outdoor activity in our study was not found after the children were divided into two groups by the age cut-off point of 12 (data not shown).

The inconsistent findings of the different studies in both Chinese urban and rural children may be attributed to the different living environments between rural and urban areas. Lin et al. reported the generational myopic shift was estimated to 1D more in Beijing urban area compared to that in Handan rural area [36, 37]. The less urbanized environment and more time outdoors were reported to be associated with lower prevalence of myopia [23, 35–37]. These may be attributed to more time spent outdoors in the sun by rural children [23, 38]. Hence, the rural children are exposed to a less myopigenic living environment compared to their urban counterparts.

There were some limitations in the present study. First, the population sample used was obtained from the offspring of the participants in the HES. Consequently, the sample size was relatively small. Second, the activities were self-reported by the children. Although this method was predominant in previously reported studies, the estimation of activity time could be subject to recall bias. However, we randomly reassessed 50 children who completed the questionnaire over a one-month period, and the weighted kappa value was acceptable (0.82) comparing the two questionnaires findings. Third, the information on activities of the children who did not respond (nearly 30%) was unknown.

In summary, in general, the association between near work and myopia was not found in this study. However, a very weak protective effect of outdoor activity on myopia in Chinese rural children was suggested. Further longitudinal studies are warranted.

## Conclusion

In summary, in this sample of rural Chinese rural children, no association between near work and myopia was found, except for children with high near work and moderate outdoor activity load. Furthermore, a weak protective effect of outdoor activity on myopia in Chinese rural children was observed.

## Additional file

Additional file 1: Dataset. (XLS 112 kb)

## Abbreviations

CI: Confidence interval
GEEs: Generalized estimating equations
HES: Handan eye study
HOMS: Handan offspring myopia study
OR: Odds ratio
STARS: Strabismus, amblyopia and refractive error study
X-PRES: Xichang pediatric refractive error study
